# Misidentifying illuminant changes in natural scenes due to failures in relational colour constancy

**DOI:** 10.1098/rspb.2023.1676

**Published:** 2023-11-29

**Authors:** Sérgio M. C. Nascimento, David H. Foster

**Affiliations:** ^1^ Physics Center of Minho and Porto Universities (CF-UM-UP), Gualtar Campus, University of Minho, 4710-057 Braga, Portugal; ^2^ Department of Electrical & Electronic Engineering, University of Manchester, Manchester M13 9PL, UK

**Keywords:** colour vision, cone-excitation ratios, colour constancy, colour relations, natural scenes

## Abstract

The colours of surfaces in a scene may not appear constant with a change in the colour of the illumination. Yet even when colour constancy fails, human observers can usually discriminate changes in lighting from changes in surface reflecting properties. This operational ability has been attributed to the constancy of perceived colour relations between surfaces under illuminant changes, in turn based on approximately invariant spatial ratios of cone photoreceptor excitations. Natural deviations in these ratios may, however, lead to illuminant changes being misidentified. The aim of this work was to test whether such misidentifications occur with natural scenes and whether they are due to failures in relational colour constancy. Pairs of scene images from hyperspectral data were presented side-by-side on a computer-controlled display. On one side, the scene underwent illuminant changes and on the other side, it underwent the same changes but with images corrected for any residual deviations in spatial ratios. Observers systematically misidentified the corrected images as being due to illuminant changes. The frequency of errors increased with the size of the deviations, which were closely correlated with the estimated failures in relational colour constancy.

## 1. Introduction

Colour signals can be ambiguous in the real world. When we view a scene under the blue of the midday sky or the yellow-orange of the setting sun, we accept that surfaces may look different but also that their reflecting properties are probably unchanged. Similar inferences can be made with other changes in lighting, not just those due to the gradual elevation of the sun [1–5], but those that are more abrupt, as when direct light is interrupted by a passing cloud or by moving foliage [1,6]. The effects may be amplified by the complex structures of natural environments [7], which can produce large temporal and spatial variations of illumination at ground level, quantified in spatial [8], directional [9,10], and time-lapse scene measurements [11–13]. The ensuing changes in appearance constitute a failure of what is usually referred to as colour constancy, that is, the constant perceived or apparent colour of a surface despite changes in the intensity and spectral composition of the illumination [14–18].

With these uncertainties in appearance, how do observers make reliable judgements about surface reflectances? Such judgements seem central to ensuring a stable visual representation of the world and veridical interactions within it. One possibility is that observers use relational colour constancy [19], which refers to the constancy of perceived colour relations between surfaces rather than of the perceived colours of the surfaces themselves. It is a weaker constancy in the sense of being necessary but not sufficient for colour constancy [19]. It has the advantage of enabling an observer ‘to correctly attribute changes in the colour appearance of a scene either to changes in the spectral composition of the illuminant or to changes in the reflecting properties of that scene [20]. This operational task is not concerned with perceptual properties such as hue and saturation but with objective events in the environment [21,22]. Observers can readily perform the task [23], with some mentioning that illuminant changes tended to be seen as a coloured wash over the display whereas reflectance changes had a more uneven appearance [20].

A quantity that could provide a physiological basis for relational colour constancy [19] is the spatial ratios of cone-photoreceptor excitations produced by light reflected from different surfaces, where the excitations are within not between long-, medium-, and short-wavelength-sensitive (L, M, and S) cone classes. More precisely, for any two points in a scene, let *r*_L_ be the ratio of L-cone excitations at the two points, and let *r*_M_ and *r*_S_ be the corresponding ratios of M- and S-cone excitations. Let **r** = (*r*_L_, *r*_M_, *r*_S_) be the resulting three-dimensional vector under the given illuminant and let $r^{\prime }=(r_{L}^{\prime },\;r_{M}^{\prime },\;r_{S}^{\prime })$ be this vector under another illuminant. Then, averaged over pairs of points, deviations between values of **r** and $r^{\prime }$ are of the order of 4% in natural scenes even with large changes in illuminant [24]. This approximate invariance in spatial ratios is maintained with ratios of linear combinations of cone signals, for example, non-opponent and opponent-colour combinations associated with achromatic and chromatic coding [25]. By definition, spatial ratios cannot provide a complete basis for colour constancy because they are independent of overall scene colour.

Although the average size of deviations in spatial ratios in scenes undergoing an illuminant change may be small, their statistical distribution generally has a long tail [24], where deviations are more noticeable. These deviations may be misinterpreted by observers as being due to reflectance changes instead of illuminant changes. Limited experimental tests of this prediction have been undertaken with the operational approach [26,27] with scenes consisting of simple regular (Mondrian) arrays of spatially uniform Munsell pigmented surfaces [28] under different illuminants. Images that were corrected for deviations in spatial ratios were systematically misidentified by observers as being the result of illuminant changes, rather than the uncorrected images.

It is not obvious, however, whether this finding with Mondrian arrays extends to natural scenes. In addition to their different spatial and spectral structures [7,8], the colour gamuts of individual scenes are typically limited, with larger variations in lightness than in chromaticity [29–35] and chromatic biases towards a yellow–blue axis [30–32,35]. A further complication is that the invariance of spatial ratios depends on the type of scene [24,36], the distance between the surfaces being compared [11], and whether there are geometrical illumination changes, as with shadows [37].

The aim, then, of the work reported here was to test the hypothesis that illuminant misidentifications occur with natural scenes and that the misidentifications are due to failures in relational colour constancy. Experimental observers viewed pairs of images of the same scene presented side-by-side on a computer-controlled display. On one side, the scene underwent illuminant changes; on the other side, it underwent the same changes but with images corrected for any residual deviations in spatial ratios. The expectation was that observers would misidentify the side with the corrected images as the one produced by illuminant changes alone. In the event, they made the expected errors, with frequency increasing with the estimated changes in colour relations.

## 2. Methods

### (a) Scenes and images

**Figure 1.**
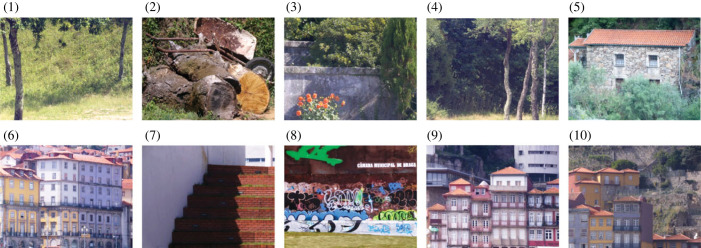
Colour images of 10 scenes used to generate the experimental stimuli. The images are rendered in sRGB format [40] from hyperspectral reflectance data with a daylight illuminant of correlated colour temperature 6500 K. The images are for illustration only. The Methods section describes how they were rendered under different illuminants in the experiment.

Colour images for the experiment were derived from hyperspectral images of 10 natural scenes chosen from a larger dataset [31,38,39], as explained shortly. They were considered natural in the sense of being part of everyday outdoor rural and urban environments, as opposed to being constructed in the laboratory, for example, as physical tableaux. Colour images of the 10 scenes are illustrated in figure 1. The choice of scenes was determined by whether they could be rendered on the monitor display (§2e), which excluded mainly those with highly saturated colours that fell outside the display gamut. This limitation contributes to a conservative test of the experimental hypothesis since in real-world scenes saturated colours produce larger deviations in spatial cone-excitation ratios and potentially greater failures of relational colour constancy [36].

Each hyperspectral image had dimensions approximately 1344 × 1024 pixels, corresponding to a camera angle of approximately 6.9° × 5.3° and spectral range 400–720 nm sampled at 10 nm intervals. The images were processed as effective spectral reflectance images so that illumination changes could be simulated by taking the product of the image with the spectra of global illuminants (§2b) to produce radiance images [36,38]. Details of the imaging system, acquisition methodology, and data processing used to derive the spectral reflectance at each pixel have been reported previously [38].

These reflectance images were smoothed by spatial averaging over 2 × 2 pixels in order to reduce non-imaging noise in the unaveraged source data [38] and pixel–pixel correlations with the 1.3-pixel line-spread function of the hyperspectral camera [41]. All images for the experiment were generated full size (excluding a narrow strip of pixels at one of the edges where a grey calibration surface was visible). They were later scaled down to 40% of their full sizes for display purposes.

### (b) Illuminants

Illumination changes were simulated with spectral changes in a global illuminant, that is, one defined by a constant, spatially uniform, spectral distribution. This approach ensures that equal changes in illumination spectrum take place at each point in the scene, isolating the role of surface reflectance from other factors such as illumination geometry [36,39,42]. The implications of incorporating the spatial and spectral variation of real illumination changes are considered in the Discussion.

To obtain a wide range of illuminant spectra with low to high correlated colour temperatures, pairs of illuminants were drawn randomly from a Planckian radiator with temperatures from an orangish 2000 K to a blueish 100 000 K. Planckian radiation is similar to daylight [2] but allows a larger range of smoothly parameterized spectra than standard models of daylight covering 4000 K to 25 000 K [43]. In practice, samples were drawn from the inverse of the Planckian temperature scale to secure an approximately uniform distribution of illuminant colours [44].

### (c) Cone excitations

Radiance images were converted to estimated L, M, and S cone excitations at each point according to the CIE 2° cone fundamentals proposed by Stockman & Sharpe [45]. All colorimetric conversions, including the calibration of the monitor display, were implemented using the CIE 2° colour matching functions [43], which are also linear combinations of the Stockman & Sharpe cone fundamentals [46]. All spectral computations were carried out with the same spectral range and resolution as the hyperspectral images.

Spatial ratios of cone excitations at pairs of points were defined as indicated in the Introduction; that is, if *q*_L_(1) and *q*_L_(2) are L-cone excitations at points 1 and 2, respectively, then their spatial ratio is given by *r*_L_ = *q*_L_(1)/*q*_L_(2), and analogously for M- and S-cone excitations, where all divisors were assumed to be positive. If $r=(r_{L},\;r_{M},\;r_{S})$ and $r^{\prime }=(r_{L}^{\prime },\;r_{M}^{\prime },\;r_{S}^{\prime })$ are the three-dimensional vectors of these ratios under two different illuminants, then a sensitive measure of their generally small differences [11, appendix A] is their relative deviation defined by $\Delta r=|r^{\prime }-r|/\min\{\left|r^{\prime }|,|r\right|\}$ where the vertical bars represent the magnitude of the vectors defined by the Euclidean norm [24,26].

Random samples of 50 000 pairs of points were drawn from the combinatorially large number of possible pairs in each image. With this sample size, the mean relative deviation (MRD) in spatial ratios $\bar{\Delta r}$ for the sample varied little across independent resamplings.

### (d) Ratio corrections

The following procedure was used to correct images for residual deviations in spatial cone-excitation ratios. For clarity, it refers to the way stimuli were produced, not how they were processed visually.

For each cone class, L say, cone excitations $q_{L}(i)$ at each point *i* under one illuminant were regressed on the corresponding cone excitations $q_{L}^{\prime }(i)$ under the other illuminant, and then replaced by their fitted values ${\hat{q}}_{L}(i)$, where ${\hat{q}}_{L}(i)$ is given by $k_{L}q_{L}^{\prime }(i)$ for some constant *k*_L_ [19]. Analogously for M and S cones. This procedure preserves spatial cone-excitation ratios exactly, since for points 1 and 2, the ratio under one illuminant $q_{L}(1)/q_{L}(2)$ is corrected to ${\hat{q}}_{L}(1)/{\hat{q}}_{L}(2)$, which is equal to $k_{L}q_{L}^{\prime }(1)/k_{L}q_{L}^{\prime }(2)$, which coincides with $q_{L}^{\prime }(1)/q_{L}^{\prime }(2)$ under the other illuminant [26, appendix].

**Figure 2.**
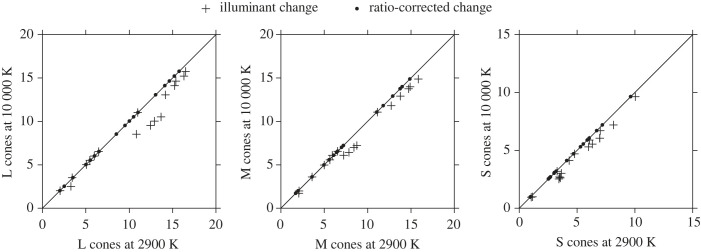
Estimated L, M, and S cone excitations from a small illustrative sample of points from scene 3 of figure 1 under a Planckian radiator illuminant with temperatures of 10 000 K and 2900 K (crosses) and with cone excitations corrected (solid circles) for deviations in spatial cone-excitation ratios. The fitted line is a linear regression. Axis scales have been adjusted for range after scaling cone excitations by the mean. See figure 3 for the images corresponding to these conditions.

Figure 2 shows a sample of cone excitations for scene 3 of figure 1 under a 10 000 K illuminant and a 2900 K illuminant, with and without ratio corrections. The fitted line is a linear regression of values of $q_{L},\;q_{M},\;q_{S}$ at 10 000 K on values at 2900 K. The crosses show how excitations deviate from the linear regression line, the largest with L cones and progressively smaller with M and S cones, as expected with the presence of reddish flowers [36]. The solid circles mark the corrected values with zero deviations for all three cone classes.

**Figure 3.**
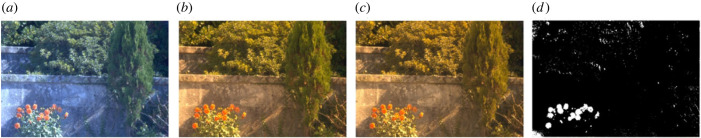
Images of scene 3 from figure 1 (*a*) under a 10 000 K illuminant, (*b*) under a 2900 K illuminant, (*c*) under a 2900 K illuminant but corrected for deviations in spatial cone-excitation ratios, and (*d*) with the locations of the largest tenth of the relative deviations marked in white.

Figure 3 illustrates the effect on appearance for scene 3 (a) under the 10 000 K illuminant, (b) under the 2900 K illuminant, and (c) under the 2900 K illuminant with ratio corrections. The differences in hue of the green foliage in (b) and (c) are perceptible but not the differences in lightness of the reddish flowers, despite larger deviations in ratios. Panel (d) marks in white where the largest tenth of the deviations are located.

Over all scenes, the MRD $\bar{\Delta r}$ in spatial ratios varied from 0% to 7%. For computational purposes, ratios were classified into bins of width 1%.

### (e) Monitor display

Images were displayed on a 31.5-inch, 1920 × 1080 pixels, 120 Hz, LCD (Display++, Cambridge Research Systems, Rochester, Kent, UK) with 14 bits per gun intensity resolution (Colour++ mode). The monitor was calibrated with a telespectroradiometer (SpectraScan PR-650, Photo Research, Chatsworth, CA). All images were displayed with an average luminance of 8 cd m^–2^ and were selected to have at least 95% of their colours within the colour gamut of the display. The software was written in MATLAB (version 9.8.0.1380330 (R2020a) Update 2, The MathWorks, Inc., Natick, MA) with the aid of the Psychophysics Toolbox Psychtoolbox (v. 3.0.18) [47].

### (f) Colorimetry

For later analysis, the colour appearance of the images was expressed within the approximately uniform colour space CAM02-UCS [43], which has coordinate *J* as a correlate of lightness, and chromaticity coordinates *a* and *b*, respectively, as correlates of redness-greenness and yellowness-blueness (notation has been simplified from the standard [43] to avoid confusion). The terms redness-greenness and yellowness-blueness refer to variables within CAM02-UCS, and should not be taken to apply outside that context.

To help establish the connection between colour relations and spatial cone-excitation ratios, sometimes taken as implicit [19,36], colour relations were assumed to be represented colorimetrically by vectors of colour differences. Thus, given a pair of points, their vector colour difference under a particular illuminant is defined by Δc=(ΔJ, Δa, Δb), as explained elsewhere [48]. If their vector colour difference under another illuminant is $\Delta c^{\prime }=(\Delta J^{\prime },\;\Delta a^{\prime },\;\Delta b^{\prime })$, then the change in colour differences can be quantified with the Euclidean norm $\Delta E=|\Delta c^{\prime }-\Delta c|$, which, although not developed here, measures the degree of generalized metamerism [39], that is, the extent to which two different colours maintain their colour difference under a change in illuminant.

**Figure 4.**
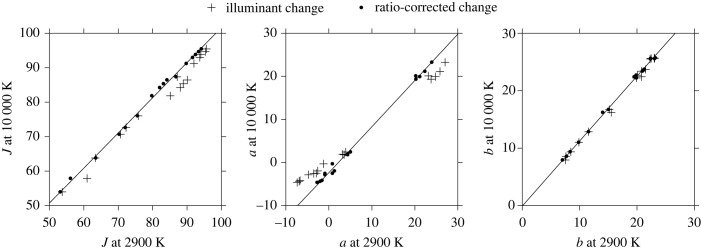
Values of appearance attributes of lightness *J*, redness-greenness *a*, and yellowness-bluenes*s b* in the approximately uniform colour space CAM02-UCS [43] corresponding to the L, M, and S cone excitations in figure 2. The fitted line is a locally weighted linear regression [49,50]. Other details as for figure 2.

To show the effects on colour appearance of correcting images for deviations in spatial ratios, figure 4 shows J, a, b values corresponding to the L, M, S cone excitations of figure 2 under 10 000 K and 2900 K illuminants, with and without corrections. The fitted line is a locally weighted linear regression [49,50] of J, a, b values at 10 000 K on values at 2900 K. Notice that the deviations in *J* values are of the same order as in *a* values, consistent with the large deviations in L-cone excitations in figure 2.

### (g) Psychophysical procedure

Observers participated in a spatial two-alternative forced-choice experiment. In each trial, two identical images of the same scene under the same illuminant, for example, image (*a*) of figure 3, were initially presented side by side. The two images then underwent an illuminant change, one without and the other with correction for deviations in spatial cone-excitation ratios, with the side chosen randomly in each trial; for example, image (*a*) was replaced by image (*b*) on the left side and by image (*c*) on the right side. The interval between image interchanges was 1 s. Four cycles of this alternating sequence were performed to allow observers enough time to look from one side to the other in order to decide which was due only to an illuminant change. After 8 s, the display was darkened and observers signalled their judgement, left or right, by pressing on a keyboard.

Before the experiment, observers were shown how a spatially uniform change with a global illuminant could be simulated with colour filters and overlays [51,52], mimicking the phenomenology of illuminant changes [20] described in the Introduction. They were not asked whether the illuminant change was natural or not [26] since the judgement risked being confounded with judgements about the larger changes in illuminant correlated colour temperature.

The viewing distance to the monitor screen was 1.2 m. Individual images subtended approximately 9° × 7° and were displayed symmetrically to the left and right of the centre of the display with a gap of 4° between them. Experiments were carried out in a darkened room, with an ambient illumination level of less than 1 lm m^–2^.

In each session, all 10 scenes were tested at each of the seven levels of MRDs $\bar{\Delta r}$ in spatial ratios, making 70 trials in all. Scenes were chosen in random order. Each session lasted for about 15 min and observers could take a break every 10 trials. In all, observers participated in 10 sessions spread across 2 to 4 days.

### (h) Observers

Five postgraduate students at the University of Minho (four female, one male, mean age 27 years) acted as experimental observers. All had normal or corrected-to-normal visual acuity and normal colour vision as assessed by the Colour Assessment and Diagnosis Test (City Occupational Ltd, Cumbria, UK) and the Heidelberger Multi-Colour-Anomaloskop (OCULUS, Inc., Wetzlar, Germany). They had a basic knowledge of colour vision and some laboratory experience mainly with clinical psychophysical colour tests but not in connection with colour constancy. They were unaware of the design and purpose of the experiment. Informed consent was obtained from all observers participating in the experiment. The experimental protocol and data handling were approved by the Ethical Committee of the University of Minho (Comissão de Ética para a Investigação em Ciências da Vida e da Saúde, CEICVS 052/2021).

### (i) Statistical analysis

Uncertainties in mean values were quantified with estimated 95% confidence intervals based on Efron's BCa bootstrap method with 1000 bootstrap replications [53]. An inverse cumulative Gaussian transformation with a variable lapsing rate [26,54] was used to fit plots of percentage misidentifications against MRDs and transformations of these quantities. A linear regression was used to fit plots of the change in colour differences within images against deviations in spatial cone-excitation ratios. A locally weighted quadratic regression [50] was also tested, with little benefit. Goodness of fit was measured by *R*^2^, the proportion of variance explained, adjusted for the degrees of freedom of the regression [55].

## 3. Results

### (a) Misidentification frequencies

**Figure 5.**
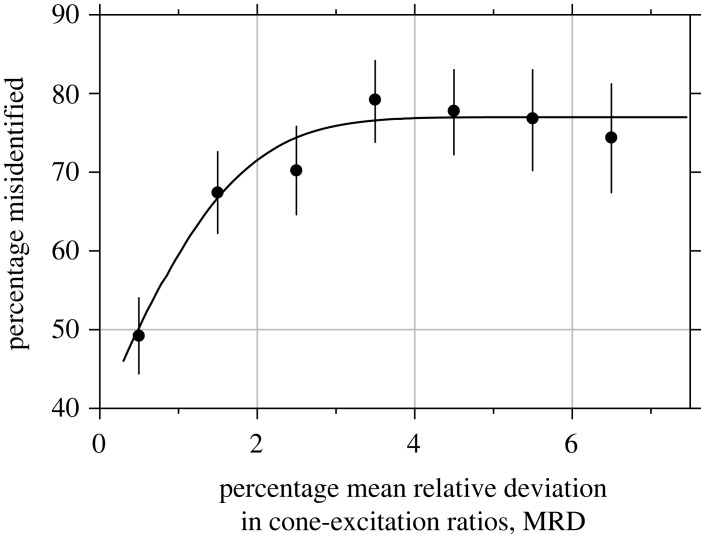
Percentage of misidentifications of illuminant changes on scenes as a function of the MRD in spatial cone-excitation ratios. Symbols show percentages averaged over observers and scene images. Error bars mark 95% confidence intervals [53]. The curve is a best-fitting inverse cumulative Gaussian transform of MRD [54]. The guessing misidentification rate was 50%.

Figure 5 shows the percentage of observers' misidentifications, that is, an ‘illuminant change’ response to images corrected for deviations in spatial cone-excitation ratios, plotted against the MRD in ratios. The percentage of misidentifications increased with MRD from chance level of 50% to around 75%. The fitted inverse cumulative Gaussian curve accounted for 92% of the variance in observers' responses. The percentage of misidentifications appears to asymptote once MRD reaches about 4%.

For comparison, observers' discrimination performance, expressed as values of the mean discrimination index *d’* from detection theory [56], was plotted against MRD, as shown in electronic supplementary material, figure S1. The pattern of performance was similar.

Observers’ descriptions of the phenomenology followed earlier accounts [20]. They reported that with uncorrected images they could see the ‘pop-out’ of elements of the scene and that less pop-out was more like a global illuminant change.

### (b) Individual scenes

**Figure 6.**
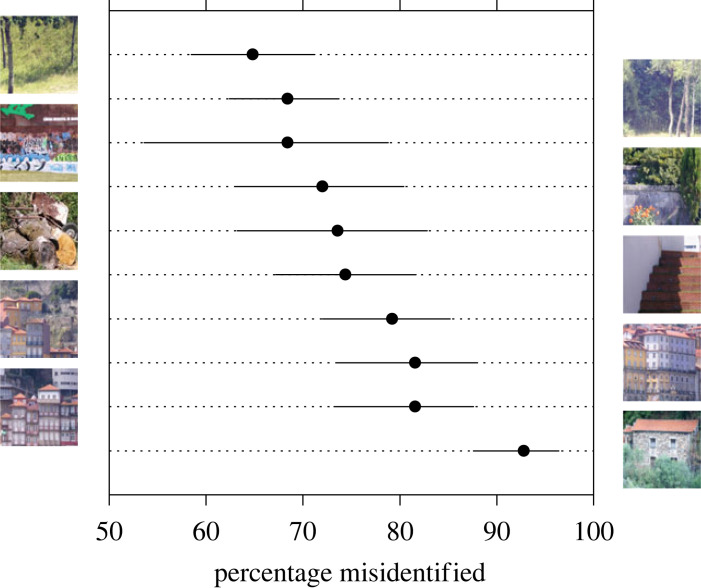
Percentage of misidentifications of illuminant changes for each scene. Symbols show percentages averaged over observers and relative deviations in spatial cone-excitation ratios from 2.5% to 6.5%. Error bars mark 95% confidence intervals [53]. The guessing misidentification rate was 50%.

Estimates of the percentages of misidentifications with individual scenes are less reliable because of the smaller sample sizes, but to indicate scene-by-scene variation, figure 6 shows percentages for each scene averaged over observers and MRDs of 2.5% to 6.5%, for which performance approached an asymptote (figure 5). All percentages were reliably higher than the guessing rate of 50%, and the highest tended to be those with scenes containing reddish reflecting surfaces [36]. Corresponding values of the mean discrimination index *d’* for each scene are shown in electronic supplementary material, figure S2. The pattern of performance was similar.

To confirm the influence of reddish reflecting surfaces in scenes, the percentage of misidentifications in each scene was plotted against the direction of the major axis of scene chromatic variation, as shown in electronic supplementary material, figure S3. There was a reliable trend for percentages to increase as the axis direction approached 0°, the reddish-greenish axis of CAM02-UCS.

### (c) Changes in colour differences

**Figure 7.**
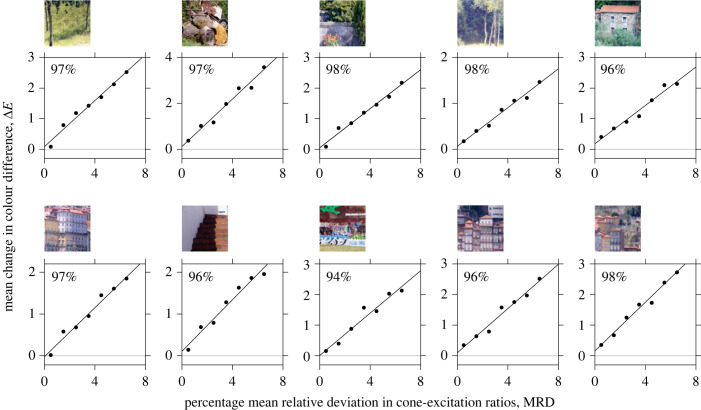
Changes in colour differences within scenes due to illuminant changes. Symbols show mean changes in estimated colour differences ΔE in the approximately uniform colour space CAM02-UCS [43] corresponding to MRDs in spatial cone-excitation ratios. Fitted lines are linear regressions with adjusted goodness of fit *R*^2^ shown in the top left of each panel. The horizontal grey line indicates zero change in colour differences.

Figure 7 shows mean changes in estimated colour differences ΔE within scenes due to illuminant changes plotted against MRDs in spatial cone-excitation ratios for samples of pairs of points. The fitted lines are linear regressions with two degrees of freedom. Values of the adjusted goodness of fit *R*^2^ range from 94% to 98%. These close correlations are consistent with deviations in spatial ratios being manifested perceptually as nonuniform illumination changes, that is, as failures in relational colour constancy [19,36]. It is stressed that these are average values and changes in colour differences across some pairs of points are larger (see figure 4). Electronic supplementary material, figure S4 shows the percentage of misidentifications of illuminant changes directly as a function of mean changes in colour differences.

## 4. Discussion

Despite the complex spatial and spectral structures of natural scenes, observers made the same kinds of errors in judging illuminant changes as they do with Mondrian arrays [26,27]. Presented with either images of scenes undergoing global illuminant changes or the same images corrected for deviations in spatial cone-excitation ratios, observers misidentified the corrected images as being due to illuminant changes. There are, though, some issues to consider before linking misidentifications to relational colour constancy.

### (a) Spatial cone-excitation ratios

The threshold for detecting changes in spatial cone-excitation ratios was of the same order of magnitude as with Mondrian arrays [26,27]. With both types of images, MRDs in spatial ratios of about 4% led to about a 75% chance of misidentifying an illuminant change. But crucially with natural scenes, the percentage of misidentifications did not increase beyond this level, by contrast with Mondrian arrays where percentages continue to increase, reaching an asymptote nearer to 90% [26]. The ceiling effect with natural scenes may be due either to their greater uncertainty [48] or to differences in the statistical distribution of deviations in spatial ratios [24]. An analysis with 50 natural scenes [36] showed that about a quarter of them had 5% of their relative deviations greater than 10%. As a side note, the detection thresholds assumed in the course of that analysis appear consistent with observers' average levels of detection recorded here (figure 5).

Although large deviations in scenes may well have attracted observers’ attention, they were free to vary their gaze and compare regions close together or far apart, presumably depending on the image structure and chromatic content [11]. Whether their viewing strategies were optimal is an open question [57].

### (b) Illumination changes

The use in this experiment of uniform changes in illumination spectrum ensured that surface reflectances were treated the same at each point in a scene [39]. Yet, as emphasized earlier, real-world changes in illumination spectrum are rarely spatially uniform and are generally confounded with geometrical changes [8,38], as the direction of the solar beam varies [1,3,4], leading to a reduction in the number of surfaces identifiable by their colour [37]. Under these conditions, spatial cone-excitation ratios are preserved only for points near each other in space and time [11]. Thus, as with the limited gamut display, the use of global illuminant changes contributes to a conservative test of the experimental hypothesis. The impact of real-world failures of relational colour constancy may therefore be underestimated.

It might still be contended that treating shaded and unshaded regions of scenes as if they were under the same illumination could inflate the extent of relational colour constancy. As a counter, masking shaded regions of images [39] has been found to have little effect on the estimated frequency of metamerism and therefore probably also on failures in relational colour constancy (§2f).

### (c) Colour relations

Extracting colour relations from scenes has both theoretical [58,59] and computational [60,61] advantages. Nevertheless, the evidence that observers use these properties to discriminate illuminant from non-illuminant changes remains indirect. As Olkkonen & Ekroll [62] noted, performance measures with the operational approach do not tell us how colour appearance changes. On the other hand, it is possible to test the converse, that is, whether candidate perceptual properties can quantitatively explain observers' performance. As shown in §3c, changes in perceived colour relations, represented as changes in colorimetric differences between pairs of points, accounted for most of the variance in observers’ misidentifications. In this sense, then, colour relations give a sufficient account of performance.

This kind of argument does not determine whether such an account is unique or minimal [63], but something similarly quantitative would be needed for any alternative explanation, for example, one proposed by Davies based on an awareness of changes in both material and lighting colour appearance [64].

## 5. Conclusion

As anticipated, observers misidentified illuminant changes in natural scenes. The frequency of misidentifications increased with the size of deviations in spatial cone-excitation ratios between surfaces. In turn, these deviations were closely correlated with the extent of failures in relational colour constancy, estimated by changes in colour appearance. Given the conservative design of the experiment, it is possible that misidentifications are at least as common in the real world, where surfaces may be more colourful and illumination changes more complex than those used here.

## Data Availability

The hyperspectral reflectance data used in this study are available at https://doi.org/10.48420/14877285 [65]. Supplementary material is available online [66].
